# “I don't opt out of things because I think I will get a sore knee, but I don't expose myself to stupid risks either”: patients’ experiences of a second ACL injury—an interview study

**DOI:** 10.1007/s00167-021-06762-x

**Published:** 2021-10-18

**Authors:** Annette Heijne, Karin Grävare Silbernagel, Mari Lundberg

**Affiliations:** 1 Department of Neurobiology, Care Sciences and Sociology, Karolinska Institutet, Stockholm, Sweden; 2 Department of Physical Therapy, University of Delaware, Newark, DE USA; 3 Department of Health Promoting Science, Sophiahemmet University, Box 5605, 11486 Stockholm, Sweden

**Keywords:** Re-rupture, ACL, Rehabilitation

## Abstract

**Purpose:**

The purpose of this qualitative study was to describe women’s experiences with anterior cruciate ligament reconstruction (ACLR) and a subsequent ACL rupture, and to identify potential facilitators and barriers for coping with rehabilitation after the second injury.

**Methods:**

Eight women between 17 and 36 years (mean 26, SD 6.5) who had experienced ACLR, followed by another ACL rupture, participated in the study. Semi-structured interviews were conducted, and data were analyzed using qualitative content analysis.

**Results:**

One overarching theme, “Rehabilitation after a second ACL injury—A lifelong adaptive coping process”, emerged from analyses. Undergoing a second rehabilitation is described as a process of adaptation, beginning with the first injury and still ongoing, more than 5 years later. Participants applied different coping strategies to adapt to these life-altering injuries, but the common denominator was of major life adjustments with no return to previous activity levels. Initially, after the reinjury, it was about coping with the catastrophe of the dreaded second injury. Over time, they accepted their “new” life and reset their recovery/rehabilitation goal not just as “return to sport” but rather as a “personal life goal”.

**Conclusion:**

Undergoing a second ACL injury is a long process that challenges the patient’s coping skills. Given these results, rehabilitation programs need to be more person centred according to the patients-adjusted life goals.

**Level of evidence:**

3.

**Supplementary Information:**

The online version contains supplementary material available at 10.1007/s00167-021-06762-x.

## Introduction

ACL rupture is the most common knee ligament injury [36], and where individuals intend returning to sport, they are usually recommended for ACL reconstruction (ACLR) and rehabilitation [23]. After these, individuals are expected to be able to return to sport [33], but the time of return to sport varies between 6 months and 27 years [22]. Long-term negative effects include reduced physical activity and quality of life (QOL) [7], and increased risk of osteoarthritis [34]. Women have a significantly higher risk of injury than men, and they experience worse patient-reported outcomes (PROs) [28] and lower return to sport rates [36].

One of the major concerns after a primary ACL rupture is to avoid a second injury. According to a recent systematic review, both sexes are at > 20% increased risk of experiencing a second ACL injury [25]. Injuries to the ipsilateral graft are more common than contralateral ACL, with ipsilateral graft injuries occurring nearly 16 months earlier after ACLR [24]. Men are incurring more injuries to the ipsilateral graft and women to the contralateral ACL [24]. Secondary ACL ruptures are associated with worse clinical and PROs than with the primary ACL rupture. Specifically, the reinjury group has a higher incidence of cartilage injuries, increased radiological changes relating to osteoarthritis, persistent and greater deficits in muscle strength in the injured leg [10, 14], worse PROs (including reduced QOL) [10, 14, 34], and lower activity levels [10]. After contralateral rupture, fewer individuals return to previous types of activity than those with a single rupture (23% vs 43%), and even fewer return to the same activity levels (12% vs 28%) [6].

Psychological factors such as self-efficacy and locus of control are linked to successful outcomes after rupture and ACLR [4, 30]. Understanding patients’ experiences of reinjury will help identify barriers to and facilitators of successful recovery, and possibly minimize negative effects on physical activity and QOL. Previous qualitative studies have looked at patients’ experiences after the first injury [17, 27, 32, 37], and there are a limited number of studies on women [17, 31]. Moreover, to our knowledge, no one has studied patients’ experiences after a second ACL. This study is to describe female athletes’ experiences of ACLR with a second ACL rupture and identify facilitators and barriers for coping with reinjury.

## Materials and methods

This was a qualitative study using semi-structured interviews and follows the Standards for Reporting Qualitative Research (SRQR). The study was approved by the Regional Ethical Board in Stockholm Registration Number 99-091, and 2013-207/32.

### Participants

These were recruited through purposive selection from a larger study comprising 68 participants who had undergone ACLR between 1999 and 2005 [16]. Included were women between 16 and 50 years with a new ACL injury within 5 years of initial surgery. Eight women met these criteria and were contacted via phone or email. Those interested received written information about the study, and semi-structured interviews were scheduled for those consenting, to be performed at locations chosen by participants. It is emphasized that all patients had twice undergone rehabilitation at a sports clinic for ACL injuries.

### Data collection

A guide, inspired by the Coping Strategies Questionnaire [26] and researchers’ clinical experience of such patients, was used to support the interview (Appendix 1).

Interviews were performed by a master’s student at Karolinska Institutet without previous contact with interviewees. The interview guide, technique and technical equipment were tested with a pilot interview (not included in results) conducted with recreational athletes who had suffered two ACL injuries, recruited from a sports clinic. The pilot was analyzed by the student and her supervisors to adjust both guide and technique.

Interviews were recorded by iPhone and MP3 player (Olympus VH-8500 PC, Digital Voice Recorder), transcribed verbatim, and analyzed according to the procedure below. The software Nvivo12 was used for coding.

### Data analysis and qualitative approach

Data were analyzed with QCA based on a general inductive approach [12, 13], meaning that category construction was data driven and no initial hypothesis guided the coding and subsequent development of categories [12]. The QCA was further divided into a combination of manifest and latent content analysis, where manifest analysis responds to a description of participants’ experiences of rehabilitation (reflected in categories), and latent content analysis responds to the underlying meaning of the text (reflected in themes) [12].

The interviews were analyzed in six steps. First, the recorded interviews and the transcribed text were reviewed until familiar with content. Second, text segments ('meaningful units’) reflecting patients’ experiences were identified. Third, meaningful units were summarized, and, fourth, condensed units were coded. The students independently read each other’s coding, resolving any disagreements. The steps were methodologically supervised by ML. In Step 5, ML with the students compared codes to identify similarities and differences, and sorted them into categories and subcategories. In Step 6, AH and the team compared the categories with the original text to ensure it correspond to participants' experiences. Thereafter, AH, ML and KGS worked iteratively to define themes and subthemes.

The trustworthiness of the analysis was confirmed by a second analysis (Steps 1–4) by ML. To increase credibility, triangulation was used during the analysis, seeking agreement among co-researchers. Representative quotations from transcribed texts are presented.

## Results

Participants were eight women aged 17–36 years (mean 26, SD 6.5). For the primary ACL rupture, five received hamstring grafts and three patellar. Subsequent ACL injuries comprised contralateral ruptures (two), ipsilateral graft ruptures (six), or bilateral (one). Times between surgeries ranged from 1 to 8 years.

### Themes and categories

One overarching theme, “Rehabilitation after a second ACL injury—A lifelong adaptive coping process”, emerged from analyses. Participants applied different coping strategies to adapt to these life-altering injuries, but the common denominator was of major life adjustments with no return to previous activity levels. Initially, after the reinjury, it was about coping with the catastrophe of the dreaded second injury. Over time, they accepted their “new” life and reset their recovery/rehabilitation goal not just as “return to sport” but rather as a “personal life goal”.

#### Category: preparation facilitates coping with a second rehabilitation

Attitudes towards rehabilitation changed between the two injuries. Although reinjury was devastating, participants agreed that the first injury was the most difficult to get through, due to lack of knowledge of the consequences of the injury and surgery, and what the rehabilitation entailed. In retrospect, they thought that more in-depth information would have facilitated rehabilitation. Already having good knowledge of the injury, the surgery and the rehabilitation created mental peace during the second rehabilitation.

**Fig. 1:**
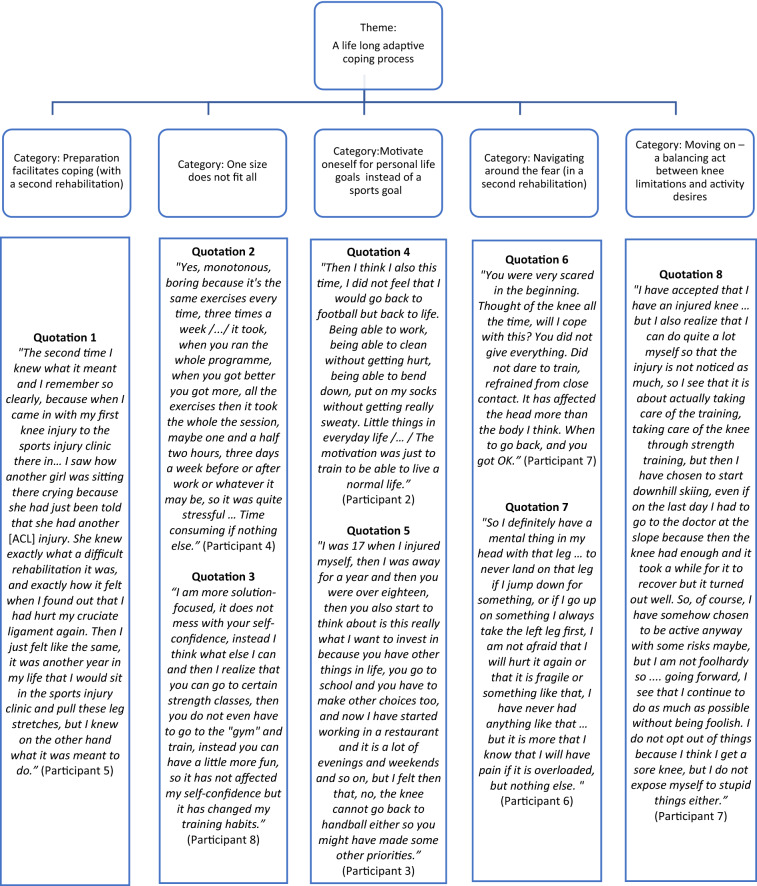
A schematic figure of the results

Quotation #1 (See Fig. 1).

#### Category: the benefit of person-centered approach (for the second rehabilitation)

Participants said the content of the rehabilitation and its organization affected their ability to cope with the second rehabilitation. Both rehabilitations were found monotonous and boring. Rehabilitation was time-consuming, making it difficult to prioritize over work and family.

Quotation #2 (See Fig. 1).

Participants made various suggestions for making rehabilitation more appealing, centered around the content, follow-up, and social support. Through participants’ stories, it is clear that one size does not fit all. Some found group rehabilitation more enjoyable and others wanted to find their own way to make training more fun.

Quotation #3 (See Fig. 1).

#### Category: motivate oneself for personal life goals, instead of a sports goal

Participants' motivation differed between the two injuries. After the first, their main motivation was to return to their previous sport and activities. Initially, after the second, they longed to return to their team and their role as an athlete but gradually, over time, the motivation changed to that of returning to a normal life.

Quotation #4 (See Fig. 1).

Experiencing an injury provides an opportunity to stop and reflect on life priorities. After the reinjury, the lengthy rehabilitation allowed them to think about what they really wanted. Leaving their sport created doubts about their identity and initiated a search for activities they liked.

Quotation #5 (See Fig. 1).

#### Category: navigating around the fear

Since their first operation, participants describe their navigation around the fear of moving with the injured knee. They experienced decreased confidence in the knee and fear of incurring a second injury. This prevented them from giving their best, and the most frequently used coping strategy was to avoid activities that risked reinjury.

Quotation #6 (See Fig. 1).

After the second injury, the fear of reinjury changed, becoming more of a risk analysis. Some completely stopped their sport activities; others adopted an active coping strategy, trying to find solutions for remaining active but, even then, there remained a nagging concern about further reinjury. They had not been able to return to previous activity levels and were still adjusting their choices of activities due to the injury. Participants continue avoiding activities they like because of the risk of losing capacities, sooner than the risk of reinjury.

Quotation #7 (See Fig. 1)*.*

#### Category: moving on—a balancing act between knee limitations and desire for activity

All participants had continuing problems with their knee(s) at the time of interview, though these were of different types. Most experienced impaired function, pain, instability and decreased strength; a few had improved stability and strength after the second operation. They described trying to accept their situation and adapt to be able to live an active life, while realizing that they have an impaired knee and cannot return to their previous level of activity.

Quotation #8 (See Fig. 1).

## Discussion

This study is the first to explore women's experiences of a second ACL injury. The most important finding was that, the participants found the rehabilitation after the first ACL injury to be worse than the second one. A well-performed prehabilitation period, and prior experiences facilitated coping with the second rehabilitation, whereas lack of guidance in returning to an active lifestyle despite injury and fear of losing capacities hindered the rehabilitation process.

The relevance of being prepared was a result in the present study and is supported by results from prior studies [15]. In other studies, following their first ACL injury, patients experienced uncertainty about what to expect after an ACL rupture and difficulty in estimating the extent of rehabilitation [5, 27]. In the last decade, prehabilitation programs have been established to better prepare patients for surgery [19]. According to evidence-based practice, prehabilitation for ACL injuries focus on reducing deficits in knee extension and quadriceps strength prior to surgery [33]. Given the results of the present study, there is no “one size fits all,” and a person-centered approach is recommended. Positive effects of a person-centered prehabilitation program have been found on health-related quality of life in patients who underwent spinal surgery [20].

Coping with fear of reinjury was a central aspect in the present interviews, and fear is also a known barrier for returning to sports after ACLR [2, 4, 6, 18, 29, 37]. Fear of movement post-injury is a normal psychological response to what individuals find threatening, and athletes are no different. Particularly interesting in our results is that, after a reinjury, fear seems to have prompted a more realistic search for activities that would not worsen their condition, as opposed to dreading a new injury. Anxiety and fear about the outcome of surgical procedures are commonly reported by patients, and an increasing number of prehabilitation programs, therefore, combine physical activity with psychological strategies [19]. Fear of movement is, however, a complex phenomenon [1, 3] and more research is needed to understand how we as health care professionals better can address and target fear in situations such as after a second ACL injury.

According to our participants return to sports was their primary goal, but over time, their goal shifted to return to an active everyday life. Filbay et al. [7] describe how individuals undergoing ACLR take several years to accept their knee function and implement appropriate lifestyle changes. Moreover, the ability to maintain a physically active lifestyle and return to sport after ACL rupture is associated with a better overall QOL [8]. As found in the present study, ACL patients need guidance in continuing an active life: physical therapists can help find things they can do, not focus on limitations. Psychologically informed practice has been successfully applied for patients with low back pain [11, 21], and might also be something to try for patients with ACL injuries. A prior qualitative study was performed on eight women after the first ACL injury [17], and three core themes representing psychosocial factors that appeared to help players cope with rehabilitation were presented: constructive communication and rich interaction with significant others, a strong belief in the importance and efficacy of one’s own actions, and an ability to set reasonable goals. These aspects should preferably be incorporated in a future rehabilitation program for ACL and recurrent ACL injuries.

Only women participated, though, notably, previous studies on ACL rupture have included predominantly male participants [28], despite women having a significantly increased risk of injury [28]. Uniquely, our study focused on female athletes: caution is advised about extrapolation to similar male athletes.

Trustworthiness is limited by the time elapsed since first injury before the interviews about that experience. Participants were asked about a rehabilitation process which might have started 10 years earlier, bringing a risk of recall bias. However, there is no other way of hearing participants’ stories. Though some details may have been missed, the data were sufficiently rich to provide us with new insights.

## Conclusions

The experience and knowledge gained from the first ACL rupture helped make reinjury and rehabilitation easier to handle. However, rehabilitation after both injuries was found boring and time-consuming. None had returned to their sport and, after reinjury, they needed support in accepting their new life situation, and guidance in returning to an active lifestyle despite injury.

## Supplementary Information

Below is the link to the electronic supplementary material.

Supplementary Appendix 1. Interview Guide (DOCX 14 KB)

## References

[1] André M, Lundberg M (2021) Thoughts on pain, physical activity and body in fearful patients with recurrent low back pain - an interview study. Accepted for publication in Physical Therapy and Rehabilitation Journal (PTJ). Personal Communication, Dr Kristin Archer June 21st, 2021

[2] Ardern CL, Webster KE, Taylor NF, Feller JA (2011). Return to sport following anterior cruciate ligament reconstruction surgery: a systematic review and meta-analysis of the state of play. Br J Sports Med.

[3] Bäck M, Caldenius V, Svensson L, Lundberg M (2020). Perceptions on kinesiophobia in relation to physical activity and exercise after myocardial infarction – a qualitative study reflecting the patients’ perspective. Phys Ther.

[4] Baez SE, Hoch MC, Hoch JM (2020). Psychological factors are associated with return to pre-injury levels of sport and physical activity after ACL reconstruction. Knee Surg Sports Traumatol Arthrosc.

[5] Burland JP, Toonstra J, Werner JL, Mattacola CG, Howell DM, Howard JS (2018). Decision to return to sport after anterior cruciate ligament reconstruction, part i: a qualitative investigation of psychosocial factors. J Athl Train.

[6] Czuppon S, Racette BA, Klein SE, Harris-Hayes M (2014). Variables associated with return to sport following anterior cruciate ligament reconstruction: a systematic review. Br J Sports Med.

[7] Filbay SR, Ackerman IN, Russell TG, Macri EM, Crossley KM (2014). Health-related quality of life after anterior cruciate ligament reconstruction: a systematic review. Am J Sports Med.

[8] Filbay SR, Crossley KM, Ackerman IN (2016). Activity preferences, lifestyle modifications and re-injury fears influence longer-term quality of life in people with knee symptoms following anterior cruciate ligament reconstruction: a qualitative study. J Physiother.

[9] Filbay SR, Ackerman IN, Russell TG, Crossley KM (2017). Return to sport matters-longer-term quality of life after ACL reconstruction in people with knee difficulties. Scand J Med Sci Sports.

[10] Gifstad T, Drogset JO, Viset A, Grontvedt T, Hortemo GS (2013). Inferior results after revision ACL reconstructions: a comparison with primary ACL reconstructions. Knee Surg Sports Traumatol Arthrosc.

[11] Godfrey E, Wileman V, Galea Holmes M, McCracken LM, Norton S, Moss-Morris R, Noonan S, Barcellona M, Critchley D (2020). Physical therapy informed by acceptance and commitment therapy (PACT) versus usual care physical therapy for adults with chronic low back pain: a randomized controlled trial. J Pain.

[12] Graneheim UH, Lundman B (2004). Qualitative content analysis in nursing research: concepts, procedures and measures to achieve trustworthiness. Nurse Educ Today.

[13] Graneheim UH, Lindgren BM, Lundman B (2017). Methodological challenges in qualitative content analysis: a discussion paper. Nurse Educ Today.

[14] Grassi A, Ardern CL, Marcheggiani Muccioli GM, Neri MP, Marcacci M, Zaffagnini S (2016). Does revision ACL reconstruction measure up to primary surgery? A meta-analysis comparing patient-reported and clinician-reported outcomes, and radiographic results. Br J Sports Med.

[15] Heijne A, Axelsson K, Werner S, Biguet G (2008). Rehabilitation and recovery after anterior cruciate ligament reconstruction: patients' experiences. Scand J Med Sci Sports.

[16] Heijne A, Werner S (2010). A 2-year follow–up of rehabilitation after ACL reconstruction using patellar tendon or hamstrings tendon graft: a prospective randomised outcome study: a prospective randomised outcome study. Knee Surg Sports Traumatol Arthrosc.

[17] Johnson U, Ivarsson A, Karlsson J, Hägglund M, Waldén M, Börjesson M (2016). Rehabilitation after first-time anterior cruciate ligament injury and reconstruction in female football players: a study of resilience factors. BMC Sports Sci Med Rehabil.

[18] Kvist J, Ek A, Sporrstedt K, Good L (2005). Fear of re-injury: a hindrance for returning to sports after anterior cruciate ligament reconstruction. Knee Surg Sports Traumatol Arthrosc.

[19] Lundberg M, Archer KR, Larsson C, Rydwik E (2019). Prehabilitation: the emperor’s new clothes or a new arena for physical therapists?. Phys Ther.

[20] Lotzke H, Jakobsson M, Brisby H, Gutke A, Hägg O, Smeets R, Lundberg M (2019). Person-centered preoperative cognitive-behavioral–based physical therapy for patients scheduled for lumbar fusion surgery—a randomized controlled trial. Phys Ther.

[21] Main CJ, George SZ (2011). Psychologically informed practice for management of low back pain: future directions in practice and research. Phys Ther.

[22] Marom N, Xiang W, Wolfe I, Jivanelli B, Williams RJ, Marx RG (2021). High variability and lack of standardization in the evalutation of return to sport after ACL reconstruction: a systematic review. Knee Surg Sports Traumatol Arthrosc.

[23] Marx RG, Jones EC, Angel M, Wickiewicz TL, Warren RF (2003). Beliefs and attitudes of members of the American Academy of Orthopaedic Surgeons regarding the treatment of anterior cruciate ligament injury. Arthroscopy.

[24] Nawasreh Z, Adams G, Pryzbylkowski O, Logerstedt D (2018). Influence of patient demographics and graft types on ACL second injury rates in ipsilateral versus contralateral knees: a systematic review and meta-analysis. Int J Sports Phys Ther.

[25] Patel AD, Bullock GS, Wrigley J, Paterno MV, Sell TC, Losciale JM (2021). Does sex affect second ACL injury risk? a systematic review with meta-analysis. Br J Sports Med.

[26] Rosenstiel AK, Keefe FJ (1983). The use of coping strategies in chronic low back pain patients: Relationship to patient characteristics and current adjustment. Pain.

[27] Scott SM, Perry MA, Sole G (2018). "Not always a straight path": patients' perspectives following anterior cruciate ligament rupture and reconstruction. Disabil Rehabil.

[28] Tan SH, Lau BP, Khin LW, Lingaraj K (2016). The importance of patient sex in the outcomes of anterior cruciate ligament reconstructions: a systematic review and meta-analysis. Am J Sports Med.

[29] te Wierike SC, van der Sluis A, van den Akker-Scheek I, Elferink-Gemser MT, Visscher C (2013). Psychosocial factors influencing the recovery of athletes with anterior cruciate ligament injury: a systematic review. Scand J Med Sci Sports.

[30] Thomee P, Wahrborg P, Borjesson M, Thomee R, Eriksson BI, Karlsson J (2008). Self-efficacy of knee function as a pre-operative predictor of outcome 1 year after anterior cruciate ligament reconstruction. Knee Surg Sports Traumatol Arthrosc.

[31] Thing LF (2006). "Voices of the broken body." The resumption of non-professional female players' sports careers after anterior cruciate ligament injury. The female player's dilemma: is she willing to run the risk?. Scand J Med Sci Sports.

[32] Truong LK, Mosewich AD, Miciak M, Pajkic A, Le CY, Whittaker JL (2021). Balance, reframe, and overcome: The attitudes, priorities, and perceptions of exercise-based activities in youth 12–24 months after a sport-related ACL injury. J Orthop Res.

[33] Van Melick N, van Cingel REH, Brooijmans F, Neeter C, van Tienen T, Hullegie W, der Sanden N-V (2016). Evidence-based clinical practice update: practice guidelines for anterior cruciate ligament rehabilitation based on a systematic review and multidisciplinary consensus. Br J Sports Med.

[34] Webster KE, Hewett TE (2021). Anterior cruciate ligament injury and knee osteoarthritis: an umbrella systematic review and meta-analysis. Clin J Sport Med.

[35] Wright RW, Gill CS, Chen L, Brophy RH, Matava MJ, Smith MV (2012). Outcome of revision anterior cruciate ligament reconstruction: a systematic review. J Bone Joint Surg Am.

[36] Zech A, Hollander K, Junge A, Steib S, Groll A, Heiner J, Nowak F, Pfeiffer D, Rahlf AL (2021). Sex differences in injury rates in team-sport athletes: a systematic review and meta-regression analysis. J Sport Health Sci.

[37] Österberg A, Kvist J, Dahlgren MA (2013). Ways of experiencing participation and factors affecting the activity level after nonreconstructed anterior cruciate ligament injury: a qualitative study. J Orthop Sports Phys.

