# Supplementary material for: “I don't opt out of things because I think I will get a sore knee, but I don't expose myself to stupid risks either”: patients’ experiences of a second ACL injury—an interview study
Source: Knee Surg Sports Traumatol Arthrosc. 2021 Oct 18;30(7):2244–50. doi: 10.1007/s00167-021-06762-x (PMC9206613; doi:10.1007/s00167-021-06762-x)
Supplement: Supplementary file 1 — Supplementary Appendix 1. Interview Guide (DOCX 14 KB) [file 167_2021_6762_MOESM1_ESM.docx]

**Appendix 1. Interview Guide**

**Theme 1. The time before the re-rupture/contra-lateral rupture**

- Describe your experiences of your knee and its function before your second cruciate ligament injury? How did you experience the stability and strength?
- Did you experience any warning signs from your knee?
- Was there anything that you experienced as an obstacle for you to be able to return to the previous activity level?
- Describe your experiences of the rehabilitation? Did you feel completely rehabilitated?
- Can you describe how you experienced your self-confidence in a sports context when you returned to sports?

**Theme 2. The time around the** **re-rupture/contra-lateral rupture**

- Describe how it happened when you injured the cruciate ligament for the second time and why you think the injury happened?

- Can you remember any special events such as associated injuries, social or psychosocial events in your life around the time of your second injury?

**Theme 3. The time after the** **re-rupture/contra-lateral rupture**

- Describe your experiences of the knee and its function after the second injury of the cruciate ligament
- How did you experience the difference between your first and second injury from a psychological perspective?
- When you had injured yourself for the second time, what choices of treatment were you faced with and how did you reason?
- How did you experience the support of your family, team members and coaches after your second injury?
- How did you experience your motivation after the operation?

**Theme 4. The future perspective**

- How do you see the future regarding your knee function? What expectations do you have on your lap?

Is there anything else that you would like to add?
